# Role stress among auxiliary nurses midwives in Gujarat, India

**DOI:** 10.1186/s12913-017-2033-6

**Published:** 2017-01-23

**Authors:** Bhaskar Purohit, Paul Vasava

**Affiliations:** 1 Indian Institute of Public Health Gandhinagar (IIPHG), Opposite Air Force Head Quarters, Near Lekawada Bus Stop, Gandhinagar Chiloda Road, Lekawada, CRPF P.O, Gandhinagar, 382042 Gujarat India; 2 Medical Officer, Department of Health, Gujarat, India

**Keywords:** Role stress, Role overload, Role stagnation, Organizational role stress, Auxiliary Nurse Midwives, India

## Abstract

**Background:**

Understanding Role Stress is important as health service providers, especially nurses experience high levels of Role Stress which is linked to burnout, poor quality of care and high turnover. The current study explicates the concept of Role Stress and assesses the Role Stress experienced by the Auxiliary Nurse Midwives (ANMs) working with rural government health centres from Gujarat, India.

**Methods:**

The study included 84 ANMs working with government health centres from one district in India. A structured instrument with established reliability and validity was used to measure 10 dimensions of Role Stress namely: Inter-role distance, role stagnation, role expectation conflict, role erosion: role overload, role isolation, personal inadequacy, self-role distance, role ambiguity and resource inadequacy. The study instrument was based on 5 point Likert rating scale that contained 50 unidirectional negative statements, 5 for each dimension. Kolmogorov-Smirnov and Shapiro-Wilk test were carried out to assess if the data were normally distributed. Cronbach’s alpha test was carried out to assess reliability of the instrument. The study data was analyzed using descriptive statistics mainly using mean scores with higher scores indicating higher Role Stress and vice versa. The data was analyzed using SPSS version 19.

**Results:**

Kolmogorov-Smirnov and Shapiro-Wilk test indicated that the data were normally distributed. Cronbach’s alpha test indicated values of 0.852 suggesting high reliability of the tool. The highest Role Stress among ANMs was experienced for resource inadequacy. Role overload, role stagnation and inter-role distance were among the other important role stressors for ANMs. The study results suggests that ANMs frequently feel that: they do not have adequate amount of resources, facilities and financial support from the high levels authorities; people have too many expectations from their roles and as result they are overloaded with work and have very limited opportunities for future growth.

**Conclusion:**

The current study has the potential to provide a useful and a comprehensive framework to understand the Role Stress among the health service providers that could be further useful in designing interventions specifically aimed at reducing Role Stress in order to prevent burnout thereby addressing the productivity and retention.

## Background

India is among the countries with critical shortages of health service providers, particularly of doctors and nurses [1]. There are considerable imbalances in how qualified health personnel are placed and distributed within India with large differences between the states as well as within the states; everywhere the distribution of health service providers favors urban areas [2]. Among health service providers, doctors and nurses currently form 50% of the total health workforce in India. However the public health sector in India particularly suffers with shortfall in the recruitment and deployment of nurses in a context where between 2007 and 2009, there was an increase in the number of nurses, midwives, and doctors in the country, but the number of vacant posts in government health facilities for these positions hardly increased [3].

Despite efforts to increase resources in the health-care sector in India, health indicators remain low and the quality of public services is generally poor [4]. Plausible reasons for poor progress of health indicators is the insufficient number of Auxiliary Nurse Midwives (ANMs)^1^ to serve in rural and remote areas. Further, work related stress experienced by Nurses has been associated with lower job satisfaction, lower organizational commitment, higher turnover intentions, and increased physical and mental health symptoms [5]. Several policy level interventions and research efforts have been made in various states of India to address the numeric inadequacy and inequitable distribution of ANMs, especially in rural areas. Such efforts have focused on addressing shortages with the introduction of various Human Resource (HR) policy interventions centred on educational and regulatory measures and on providing hardship allowances for providers serving in remote areas [6]. Despite such efforts, many states struggle to fill up the vacant positions in rural health centres. This paper focuses on the need to understand the role related stress experienced by ANMs as it has a strong relationship with motivation, job satisfaction and burnout [7]. Understanding stress associated with work may be useful to design interventions to attract and motivate health service providers to work in underserved areas and to ensure quality of care.

Work-related stress is a global issue [8]. However nurses are particularly vulnerable to work related stress as today the health systems and nursing workforce face limited resources and increasing demands on their services [9] and the nurse managers are required to work in increasingly complex environment which makes them vulnerable to stress owing to increased job demands [10, 11]. According to the US institute of Occupational Health and Safety, nursing is among the top professions with highest prevalence of stress related disorders [12, 13]. “Nursing is, by its very nature, an occupation subject to a high degree of stress [14]”.

Globally, among various sources of stress among nurses midwives, work overload is a main one [15] and if prolonged exposure to stress is not handled properly, it may lead to burnout [16, 17]. Burnout in turn affects quality of care, and is associated to higher absenteeism and increased turnover [18, 19]. While some causes of stress are related to the clinical work of the nurses, stress is also a result of the role and organizational pattern within which they work [20]. Hence with the shortage of ANMs in India and with the challenging and complex nature of their work, it becomes important to study the stress ANMs experience as a result the roles they play. Better understanding of ANMs roles and role related stress may help design interventions that address such stress factors by preventing burnout and improving job satisfaction among ANMs, thereby increasing their retention and scaling-up quality of care. With this backdrop the current study aimed at assessing the overall role stress and ten different types of role stress experienced by ANMs.

### The concept of role stress (RS)

“Stress can be defined as a dynamic condition in which an individual is confronted with an opportunity, constraint, or demand related to what he or she desires and for which the outcome is perceived to be both uncertain and important” [21]. Stress can be of various types and many factors such as personal, familial and personality type can predispose an individual to stress [22]. While individuals may experience stress as a result of several factors, one of the most important kinds of stress experienced by health service providers is the ‘role related stress’. Three kinds of role related stress: role overload, role ambiguity and role conflict have been found to be the main antecedents of burnout [23]. Further, role ambiguity and role conflict have been described to be inherent in nursing roles [24].

The concept of role is defined as the set expectations various people called significant others,^2^ including the role occupant have in relation to a position and how the role occupant responds to such expectations [25]. Since the concept of role is defined by expectations, stress is almost inevitable. The concept of role is important as it is through the roles that individuals and organisations interact [26]. Therefore role stress can be defined as the stress experienced as a result of incompatibility among various expectations of significant others and the role occupant have in relation to a specific role [25]. Role Stress (RS) is the stress resulting from the organizational role and the stress on the role occupant in being able to perform or not being able to perform the role [26]. While the concept of job is more prescriptive in nature, role includes more discretionary part of work.

An important approach to study Role Stress is to understand stress resulting as a lack of fit between a person (in terms of personality, aptitudes, and abilities) and the work environment, along with a consequent inability to cope with the various demands the role imposes on role occupant [27]. Stress resulting from the occupation of an organizational role and the ability to perform or not perform it explains the Organizational Role Stress (ORS) or Role Stress [26] which has been studies in detail in the current study. ORS is experienced as a result of incompatibility between various expectations of Significant others and the role occupant have from the role in question.

Pioneering work on role related stress by Kahn et al. identified three dimensions of organizational role stress: *role conflict, role ambiguity,* and *role overload* [28]. Scales for measuring role conflict, role ambiguity [29] and role overload were later developed which were criticized on the grounds of low validity [30, 31]. While despite the complexities involved in the concept of assessing role related stress, the focus of research was primarily only on three aspects of role stress: role conflict, role ambiguity, and role overload. Later Pareek identified eight types of organizational role stress that closely represented most problems encountered in organizational roles [32] and designed an instrument called Organizational Role Stress (ORS) that captured ten types of Role Stress that are used in the present study [26]. Hence Pareek’s work on Role stress and the instrument to assess Role Stress developed by Pareek which is used in the currently study, is very relevant to the study and advances the pioneering work on Role Stress carried out by Kahn et al.

### Conceptual basis of the study instrument

An organization encompasses many systems of roles. From the individual’s point of view, there are two role systems. The first is the system of various roles that the individual performs, such as doctor, parent, sibling or any other social role: this is role space. At the centre of the role space is the self. Which refers to cognitive structure that evolves from past experience with other persons and objects. An individual performs various roles some of which may be centered around or closer to the self while others could be at varying distances from the self. These relationships define the role space, which is the dynamic interrelationship between the self and the various roles an individual occupies. Any incompatibility or incongruence between the self and various roles that the individual occupies results in various kinds of role stress [25].

The second is the role set or the system of various roles in the organization in which the role of individual is inserted. The role set is a pattern of interrelationships between the role in question (ANMs in the current study) and among other roles in the organization. In a role set map, the focal role is in the centre. Any incompatibility or incongruence between the focal role and various other roles that are significant to the focal role results in various kinds of stress [25]. Based on the two role sets, i-e role space and role set, ten dimensions of Role Stress were studied in the current research. The conceptual framework for the study has been adapted from the work of Pareek of Role Stress [27].

- Inter-role distance is a kind of stress that arises when the role occupant is unable to balance between two or more of the roles. This is because a role occupant plays more than one role and if one of these roles is excessively demanding then the other roles may suffer leading to stress.
- Role stagnation is due to lack of opportunities for growth.
- Role expectation conflict is experienced if there are conflicting expectations from the role senders^3^ or significant others.
- Role erosion: Stress related to role erosion is experienced when the credit for one’s accomplishment in the role is given to some other person in another role or some important function belonging to one’s role is performed by some other person in another role.
- Role overload is experienced on having either too many or too high expectations from a role. It may be defined as inability to fulfill organizational expectations in time available [33].
- Role isolation exists when the role occupant does not have the required frequency or ease of interaction with others in her role set, this sort of role stress is experienced.
- Personal inadequacy is a kind of role stress which is experienced when the role occupant lacks the necessary knowledge, skills, or experience needed for the work.
- Self-Role Distance is experienced when the role occupant does not find his role interesting and motivating or when the role occupant’s special skills remain unutilized, or when there is a conflict between role occupant’s image/needs/values and those of his role.
- Role ambiguity related stress is experienced when the role occupant is not clear about expectations from the role or the role occupant doubts that certain responsibilities, functions, or activities are part of his/her role. Role ambiguity may also be defined as lack of specificity and predictability concerning an employee’s job and responsibilities [28].
- Resource inadequacy is experienced if the resources to the role occupant are not available for carrying out the role responsibilities.

Role Stress is an important concept which has received little attention from researchers in the public health sector in India [7, 34]. While there is a fair amount of literature available on Role Stress and burnout among nurses outside India, there is very limited literature available on Role Stress experienced by public health sector Auxiliary Nurses-Midwives (ANMs). Research on Role Stress becomes important as a few role stressors included in the current study have been found to be significant contributors in burnout among nurses in studies within and outside India [35].

## Methods

The data for the study was collected during April to June 2012 from one district in Gujarat state which is a Western state in India. The study district was purposively selected as one the researchers of the study was familiar with the district and the district authorities and this made the data collection and administrative procedures for the study smoother. The density of health service providers,, nurses and midwives working in Gujarat, is 2.89 and 2.12 per 1000 population respectively while the ratio of nurse and midwives per doctor was 2.77 [3]. The selected district for the study comprised of five blocks.^4^ The quantitative survey tool was administered with ANMs working with the Primary Health Centres (PHCs)^5^ and Sub Centres (SCs)^6^ from the study district.

Stratified sampling technique was used for selection of ANMs. The state health department has graded all PHCs in the district from A to D based on performance indicators with A as the best grade and D the worst grade. Inclusion of ANMs in the study was based on stratification of the graded facilities to ensure that there was representation of ANMs from all blocks as well as all the graded health facilities based on performance. Further all the ANMs working in the selected PHCs and all the SCs were included in the study. In total 84 ANMs were included in the study that represented 13 PHCs and 60 SCs from all the five blocks of the selected district.

The Organizational Role Stress (ORS) instrument was used for the data collection developed by Pareek [27]. The ORS tool provides information on ten dimensions of role related stress which were as follows: Inter-role distance, role stagnation, role expectation conflict, role erosion: role overload, role isolation, personal inadequacy, self-role distance, role ambiguity and resource inadequacy. The ORS instrument has been widely used in India outside health sector [36, 37]. The ORS instrument has also been used in the health sector in India mostly with nurses [7, 34, 38, 39]. The use of tool in the health sector is not limited to India but the same tool has also been used outside India to assess role stress among public nurses in Philipines [35]. The ORS has been identified as a classic tool for measuring ORS [40]. The tool has established reliability and validity [41]. The ORS instrument is based on 5 point Likert rating scale from 0 to 4 for each statement where higher scores indicate greater role stress and vice versa. The instrument contained 50 unidirectional negative statements, 5 for each of the ten dimensions. Since there were 5 statements of each dimension, the average scores for each dimension could theoretically range from 0 to 20 with higher scores indicating higher Role Stress and vice versa.

Kolmogorov-Smirnov and Shapiro-Wilk test were carried out to assess if the data were normally distributed. To assess reliability of the instrument and to assess inter item correlation among the 10 types of role stress, Cronbach’s alpha test was carried out. The study data was analyzed using descriptive statistics. Since there were 5 statements for each type of role stress assessed in the study, average scores were computed for the ten types of role stress that theoretically could range from 0 to 20 with higher scores indicating higher Role Stress and vice versa. The data was collected by one of the researchers of the study by visiting each respondent individually. The data was analyzed using SPSS version 19 [42].

The ethical approval for the study was sought from institutional ethical review committee at Indian Institute of Public Health Gandhinagar (IIPHG). Informed written consent of the respondents was taken before data collection. The participation in the study was completely voluntary and respondents were assured of anonymity at all times of the study. Necessary permission for the study was taken from appropriate district authorities.

## Results

The demographic profile of the study respondents is presented in Table 1. Next in the results section, the results related to Kolmogorov-Smirnov and Shapiro-Wilk are discussed which suggest whether the study data was normally distributed or not (see Table 2). Finally in results section the overall role stress scores as well as scores related to ten dimensions of role stress are presented in Table 3.

**Table 1:** Details of ANMs included in the study

Blocks	ANMs (%)
Block 1	14 (17%)
Block 2	32 (38%)
Block 3	18 (21%)
Block 4	9 (11%)
Block 5	11 (13%)
Total	84
Health Facilities	
SC	60 (71%)
PHC	24 (29%)
C.H.C.	0
D.H.	0
Total	84
Mean Age (in years)	43.1
Average Work Exp (in Years)	16.9

**Table 2:** Test to assess normality of data

Respondent	Kolmogorov-Smirnov			Shapiro-Wilk		
	Statistic	df	Sig.	Statistic	df	Sig.
ANM	.072	84	.200	.978	84	.170

**Table 3:** Mean, Median and SD Scores for different dimensions of RS for ANMs

No.	Dimensions	ANMs (*N* = 84)		
		Mean	Median	SD
1	Inter role distance	10.86	11	3.68
2	Role stagnation	11.65	12	3.91
3	Role expectation conflict	7.26	8	3.46
4	Role erosion	9.98	10	3.56
5	Role overload	12.05	13	3.74
6	Role isolation	8.25	9	3.31
7	Personal inadequacy	9.7	9	3.89
8	Self-role distance	7.44	7	3.42
9	Role ambiguity	9.07	9	5.48
10	Resource inadequacy	12.26	13	3.93
	Overall Role Stress	9.86	9	2.44

Table 1 explains that of the total 84 ANMs included in the study, 17% (14) were from block 1, 38% (32) were from block 2, 21% (18) were from block 3, 11% (9) were from block 4 and 13% (11) were from block 5. As far as place of work is concerned, 71% (60) worked with the SCs while 29% (24) worked with PHCs. The average age of ANMs it was 43 years while the average year of work experience for ANMs was approximately 17 years. (For details please see Table 1).

Since the study data to assess role stress was based on Likert scale, Kolmogorov-Smirnov and Shapiro-Wilk test were carried out to determine if the data were normally distributed. Both the test indicate that the data were normally distributed with a significance level of .200 using Kolmogorov-Smirnov test while significance level of .170 for using Shapiro-Wilk test. (See Table 2 for details).

In order to assess the reliability of the instrument and to assess inter item correlation among the 10 types of role stress, Cronbach’s alpha test was carried out and indicated a values of 0.852 suggesting high reliability of the tool.

Table 3 explains the mean and median scores for then types of role stress as well as scores for overall role stress which has been calculated by combining the scores for ten types of role stress. Since the mean and median scores for overall role stress as well as for ten types of role stress were nearly the same, it can we concluded that the data was normally distributed. The results indicate that ANMs experience the highest stress related to Resource Inadequacy (12.26) followed by role overload (12.05) and then role stagnation (11.65) while the lowest stress is experienced in relation to role expectation conflict with a score of 7.26. The overall mean for Role Stress score for ANMs was 9.86 (see Table 3 for details).

## Discussion

This was an exploratory study that aimed at assessing the role stress experienced by ANMs in public health services in Gujarat, India.

The present study found role overload to be one of the main stressors for study respondents indicating that ANMs experience high demands, possibly exceeding their abilities, from significant others. This finding is supported by some national and international literature. In a study of nurses in India, workload was found to be a key factor to take into account in developing effective Human Resource for Health (HRH) strategies such recruitment and retention for nurses [43]. Another study on nurses in a government hospital in India found role overload to be the highest stress contributing factor [39]. Other studies in India reported that role overload of nurses was higher than in other professions [44], possibly because long working hours were part of the work culture [45] and that heavy workload and work-family conflict were key turnover factors [38]. Similarly a study of the first line nurse managers in Sweden reported greater workloads [46]. Hence from the point of view of Human Resource Management (HRM), it is important to focus on understanding role overload as it has also been identified as a significant predictor of burnout among nurses in a general hospital in Spain [13]. Imbalances in the work and personal life - insecure work, high workload and pressure at work, can cause problems that employees carry over into their personal lives, and can have a negative impact on their well-being [45]. Furthermore a study with nurses from Canada found that the work related stress experienced by the nurse managers is induced by role overload [47].

Resource inadequacy or lack of resources was found to be the most significant role stressor for ANMs. The high scores for resource inadequacy indicates that ANMs often felt that they do not have the resources as well as the power to make decisions in their work. Lack of resources and powers to make decisions have also been found to be stressors among health professionals in university and state level hospitals in Turkey [48]. Similarly another study done with nurse managers from Canada found that inadequate human resources, which in some sense reflects lack of adequate resource was lined to work related stress [47].

The study also found that in the particular case of ANMs, role stagnation was an important stressor. Respondents reported a feeling of being stuck in the same role as there are few opportunities for personal growth and advancement. Role stagnation or lack or professional growth and opportunities have been found to be important turnover factors [38, 49], particularly among nurses [50]. Hence efforts must be made to provide needed resources to ANMs to carry out their roles more effectively. Failure to do so may result in higher stress which may further lead to burnout and reduced job satisfaction affecting turnover and productivity negatively.

Although the study found that the mean scores for role ambiguity were not as high as other role stress dimensions, it indicates that it is an important dimension of role stress which requires the attention of managers as role ambiguity has been found to be an important predictor of depersonalization, lowered personal accomplishment and emotional exhaustion among Indian nurses [50], as well as a significant predictor of burnout among nurses from a general hospital in Spain [13].

High scores for the dimension of inter-role-distance indicate that the ANMs experience conflict between their organizational and non-organizational roles that may lead to lower engagement in their work and reduced organizational commitment. A study from India found that high inter-role-distance scores to be higher among groups such as nurses than in other professions whose roles involve more emotional work [50]. High role stress under inter-role distance suggest an imbalance in two or more roles and has been found to be one of the highest stress contributing factors [39].

The study has several limitations. Firstly, it covered a small group of ANMs with small geographical coverage, which limits external validity. Secondly, it is a descriptive study of the different role stressors experienced by the health service providers. Future research should explore further the correlation between role stress and burnout and job satisfaction. Thirdly, there were no norms available for cut off scores for both high and low scores for the different role stress dimensions; in the absence of such norms, it was only possible to report average scores. Lastly, the study reports perceived stress which may be different from actual physiological stress experienced by health service providers. Despite these limitations, the study has the potential to provide a useful framework and instrument to understand and assess the role related stress among ANMs. Such a framework becomes particularly important in light of limited research on the topic in the complex and dynamic health services systems where ANMs have to constantly adapt to changes. The study found that resource inadequacy, role overload and role stagnation were the main role stressors for ANMs.

## Conclusion

The study used a comprehensive framework to assess Role Stress among health service providers i-e- ANMs from public sector in India. Resource inadequacy, role overload and role stagnation were found to be the main role stressors with high mean scores for ANMs. The study results indicate that ANMs are prone to organizational role stress. The findings reiterate that nurses experience role stress in relation to role overload and role stagnation but also that other role stress factors warrant research attention. The study is one of the first attempts in Gujarat, India to suggest important role stressors beyond role ambiguity and overload which have been more commonly studied. Adequate strategies to manage role stress may reduce burnout and increase job satisfaction which may in turn improve quality of care and retention of ANMs. However in the absence of any norms for role stress scores in public health sector, it is difficult to assess and comment on whether role stress scores obtained in the study are high or low. There is a need for research to develop some norms of desirable or acceptable levels of role stress scores for its different dimensions.

## Abbreviations

ANM: Auxiliary Nurse Midwives
CHC: Community Health Centre
DH: District Hospital
ORS: Organizational Role Stress
PHC: Primary Health Centre
RS: Role Stress
SC: Sub Centre

## Footnotes

1 Auxiliary nurse midwife (ANM) is a village-level female health service provider/worker in India who is known as the first contact person between the community and the health services. ANMs are the front line health care providers closest to the community who make home visits and provide Maternal and Child Care to women in their allotted villages. In addition ANMs are multi-purpose health workers and their work includes maternal and child health, family planning services, health and nutrition education, immunization for the control of communicable diseases, treatment of minor injuries etc. along with conducting deliveries. ANMs undergo a two year to two and a half year diploma training programme and require a minimum of 12^th^ grade education.

2 Significant others may be defined as all those roles that are important to the role in question and have expectations from the role in question. For example for the role of ANM, a doctor from the health center and community are Significant others as they have expectations from the role in question which is ANM.

3 Role senders are those people who have expectations from the role in question.

4 A block is a large administrative subunit of a district comprising of many villages

5 A PHC covers a population of 20,000 in hilly, tribal, or difficult areas and 30,000 populations in plain areas with 4–6 indoor/observation beds. It acts as a referral unit for 6 sub-centres and refer out cases to CHC (30 bedded hospital) and higher order public hospitals located at sub-district and district level.

6 Sub Centre (SC) is the most peripheral and first contact point between the primary health care system and the community. A SC is manned by one Auxiliary Nurse Midwife (ANM) and one multipurpose worker. A SC caters to a population between 3000 and 5000.
